# Supplementary material for: Unveiling Sex-Based Differences in the Effects of Alcohol Abuse: A Comprehensive Functional Meta-Analysis of Transcriptomic Studies
Source: Genes (Basel). 2020 Sep 21;11(9):1106. doi: 10.3390/genes11091106 (PMC7564639; doi:10.3390/genes11091106)

Figure S4. UpSet plots showing the number of common and specific KEGG pathways in women (a) and men (b).

a

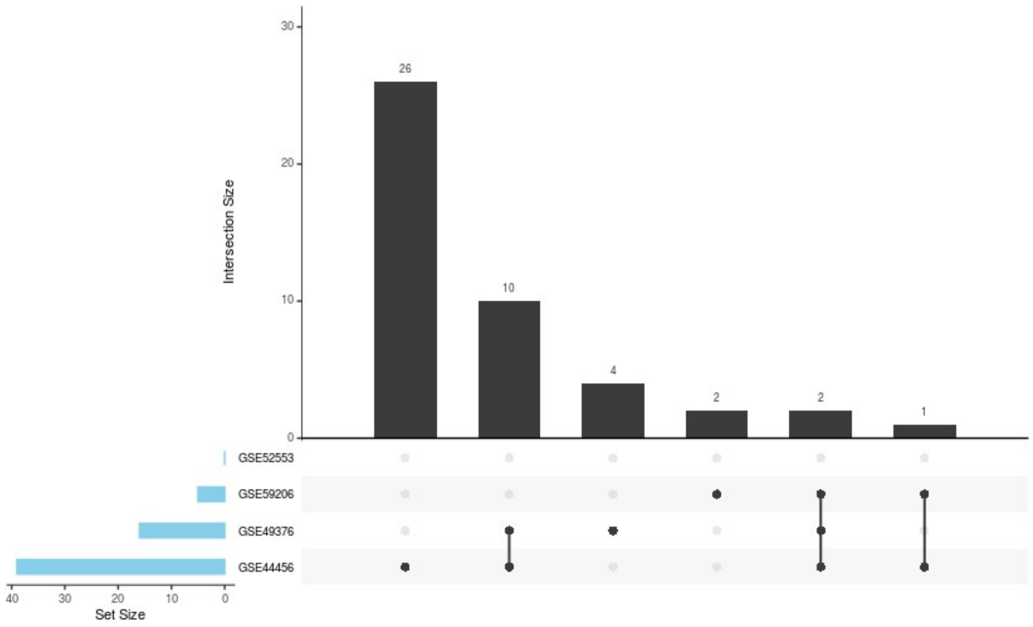

b

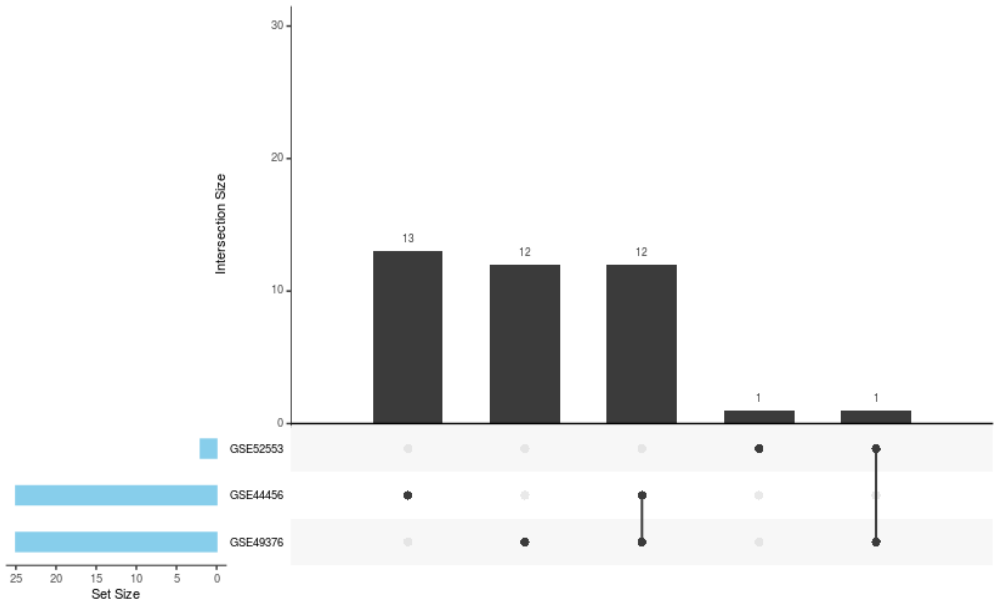

Supplement: Supplementary file 1 [file genes-11-01106-s001.zip › FigureS4.pdf]
