# Supplementary material for: Unveiling Sex-Based Differences in the Effects of Alcohol Abuse: A Comprehensive Functional Meta-Analysis of Transcriptomic Studies
Source: Genes (Basel). 2020 Sep 21;11(9):1106. doi: 10.3390/genes11091106 (PMC7564639; doi:10.3390/genes11091106)
Supplement: Supplementary file 1 [file genes-11-01106-s001.zip › FigureS5.pdf]

Figure S5. Summary of main functional groups of cellular components by sex. Each treemap depicts the significant functions overrepresented in women (a) and men (b). Treemaps are organized into two levels: the first level visualizes the most general functional groups in Gene Ontology hierarchy as large rectangles, while the second level represents the significant cellular components that integrate each functional group by small rectangles of the same color.

a

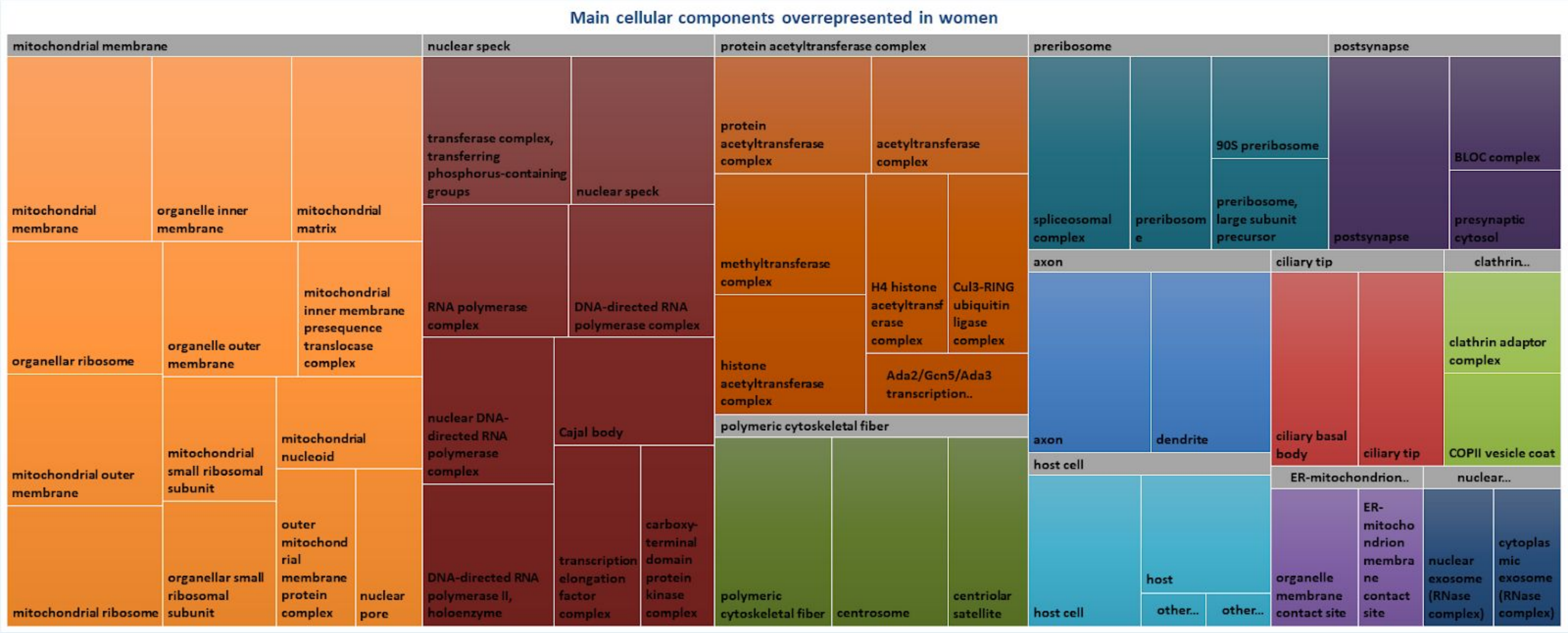

b

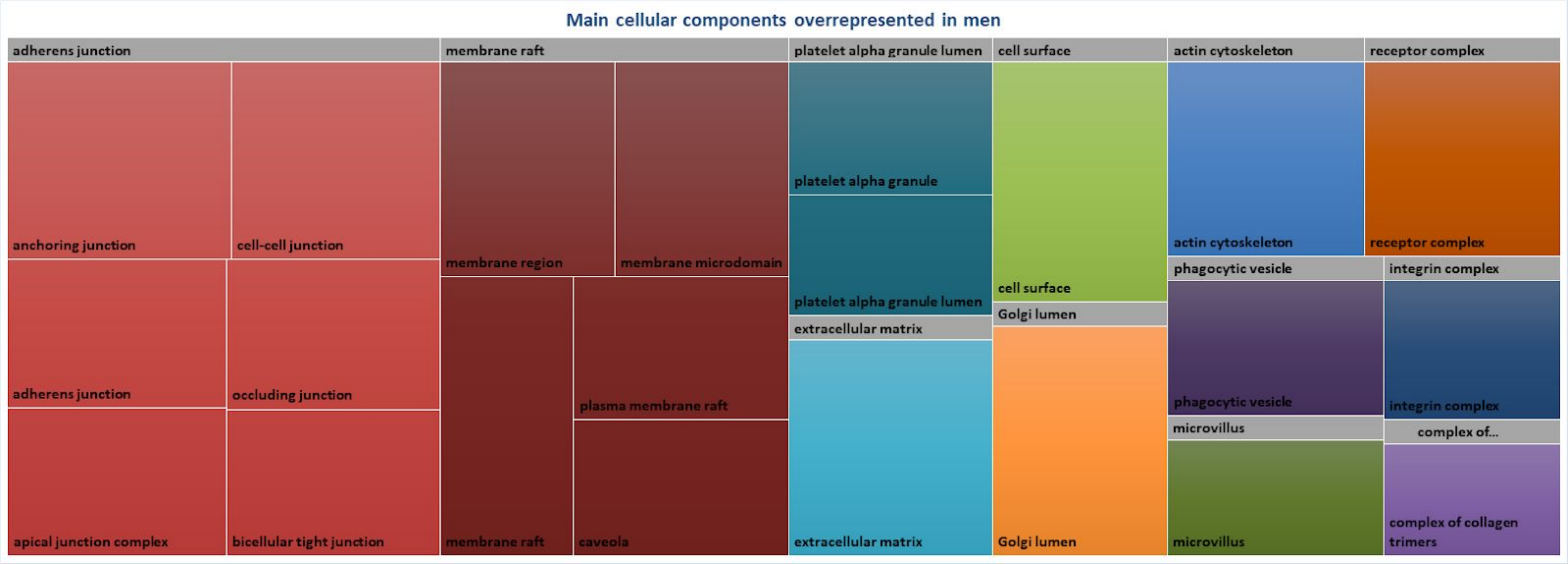

Main cellular components overrepresented in men
adherens junction
anchoring junction
cell-cell junction
adherens junction
occluding junction
apical junction complex
bicellular tight junction
membrane raft
membrane region
membrane microdomain
plasma membrane raft
membrane raft
caveola
platelet alpha granule lumen
platelet alpha granule
platelet alpha granule lumen
extracellular matrix
extracellular matrix
cell surface
cell surface
Golgi lumen
Golgi lumen
actin cytoskeleton
actin cytoskeleton
receptor complex
receptor complex
phagocytic vesicle
phagocytic vesicle
integrin complex
integrin complex
microvillus
microvillus
complex of...
complex of collagen trimers
