# Supplementary material for: Unveiling Sex-Based Differences in the Effects of Alcohol Abuse: A Comprehensive Functional Meta-Analysis of Transcriptomic Studies
Source: Genes (Basel). 2020 Sep 21;11(9):1106. doi: 10.3390/genes11091106 (PMC7564639; doi:10.3390/genes11091106)
Supplement: Supplementary file 1 [file genes-11-01106-s001.zip › FigureS6.pdf]

Figure S6. Summary of main functional groups of molecular functions by sex. Each treemap depicts the significant functions overrepresented in women (a) and men (b). Treemaps are organized into two levels: the first level visualizes the most general functional groups in Gene Ontology hierarchy as large rectangles, while the second level represents the significant molecular functions that integrate each functional group by small rectangles of the same color.

a

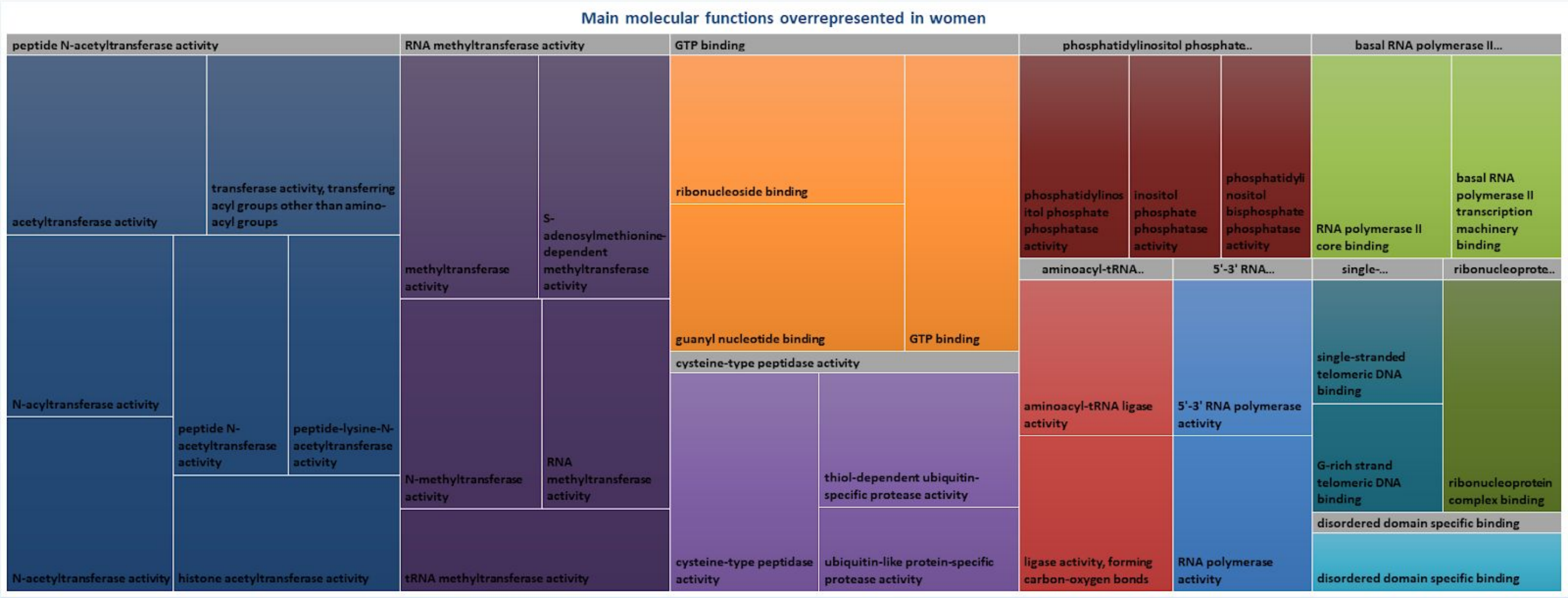

b

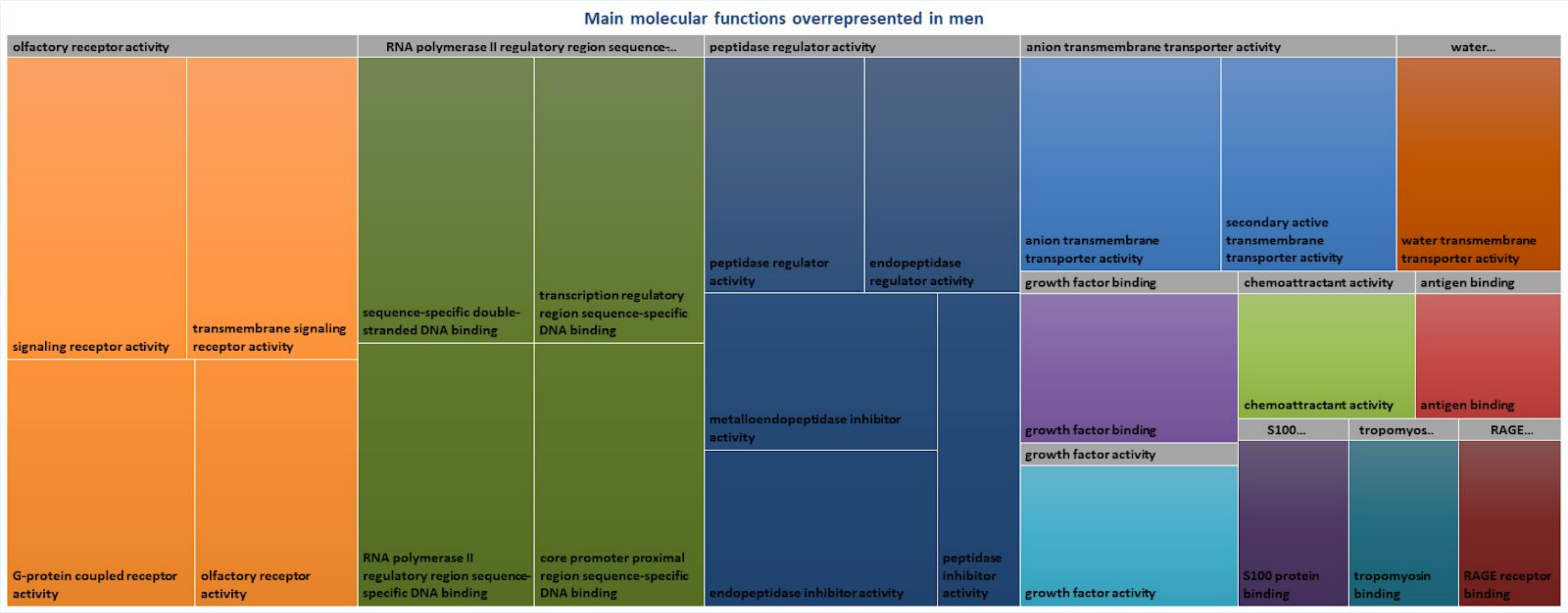
Main molecular functions overrepresented in men
olfactory receptor activity
signaling receptor activity
transmembrane signaling receptor activity
G-protein coupled receptor activity
olfactory receptor activity
RNA polymerase II regulatory region sequence-...
sequence-specific double-stranded DNA binding
transcription regulatory region sequence-specific DNA binding
RNA polymerase II regulatory region sequence-specific DNA binding
core promoter proximal region sequence-specific DNA binding
peptidase regulator activity
peptidase regulator activity
endopeptidase regulator activity
metalloendopeptidase inhibitor activity
endopeptidase inhibitor activity
peptidase inhibitor activity
anion transmembrane transporter activity
anion transmembrane transporter activity
secondary active transmembrane transporter activity
water...
water transmembrane transporter activity
growth factor binding
growth factor binding
chemoattractant activity
chemoattractant activity
antigen binding
antigen binding
growth factor activity
growth factor activity
S100...
S100 protein binding
tropomyos..
tropomyosin binding
RAGE...
RAGE receptor binding
