# Supplementary material for: Unveiling Sex-Based Differences in the Effects of Alcohol Abuse: A Comprehensive Functional Meta-Analysis of Transcriptomic Studies
Source: Genes (Basel). 2020 Sep 21;11(9):1106. doi: 10.3390/genes11091106 (PMC7564639; doi:10.3390/genes11091106)

Figure S7. Summary of main functional groups of biological processes by sex. Each treemap depicts the significant functions overrepresented in women (a) and men (b). Treemaps are organized into two levels: the first level visualizes the most general functional groups in Gene Ontology hierarchy as large rectangles, while the second level represents the significant biological processes that integrate each functional group by small rectangles of the same color.

a

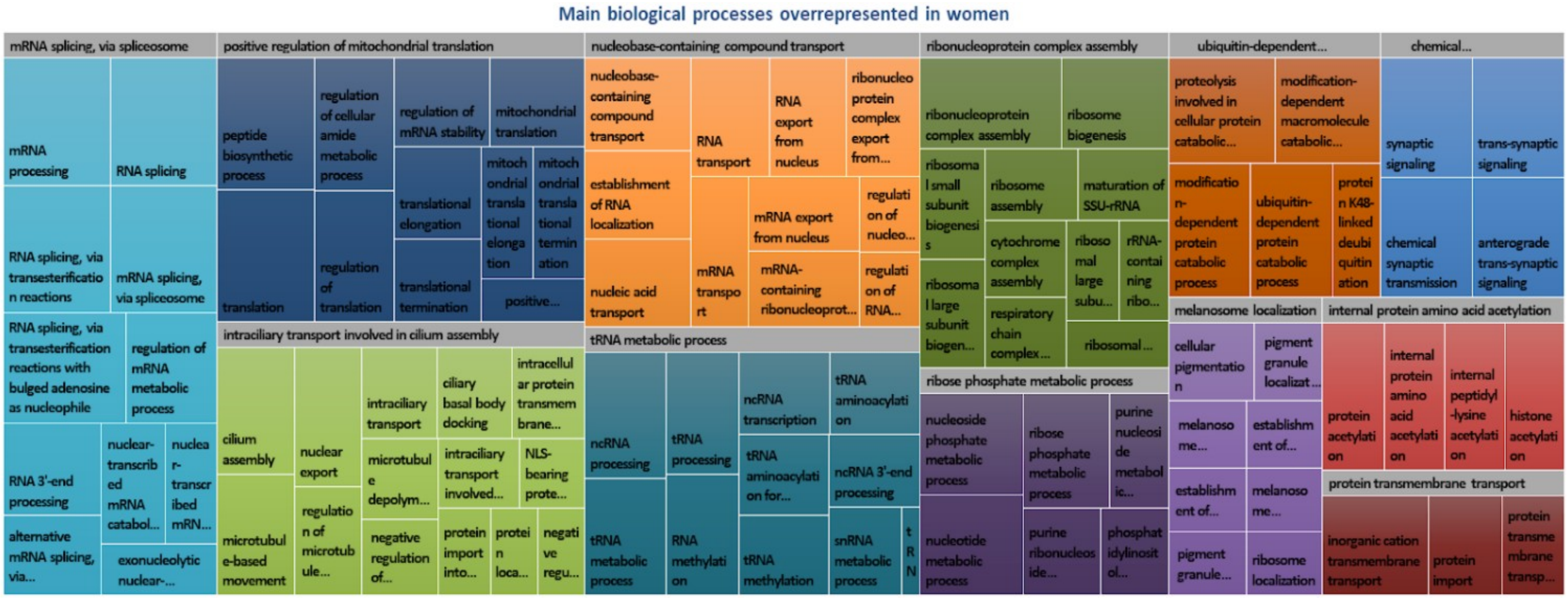

b

Main biological processes overrepresented in men

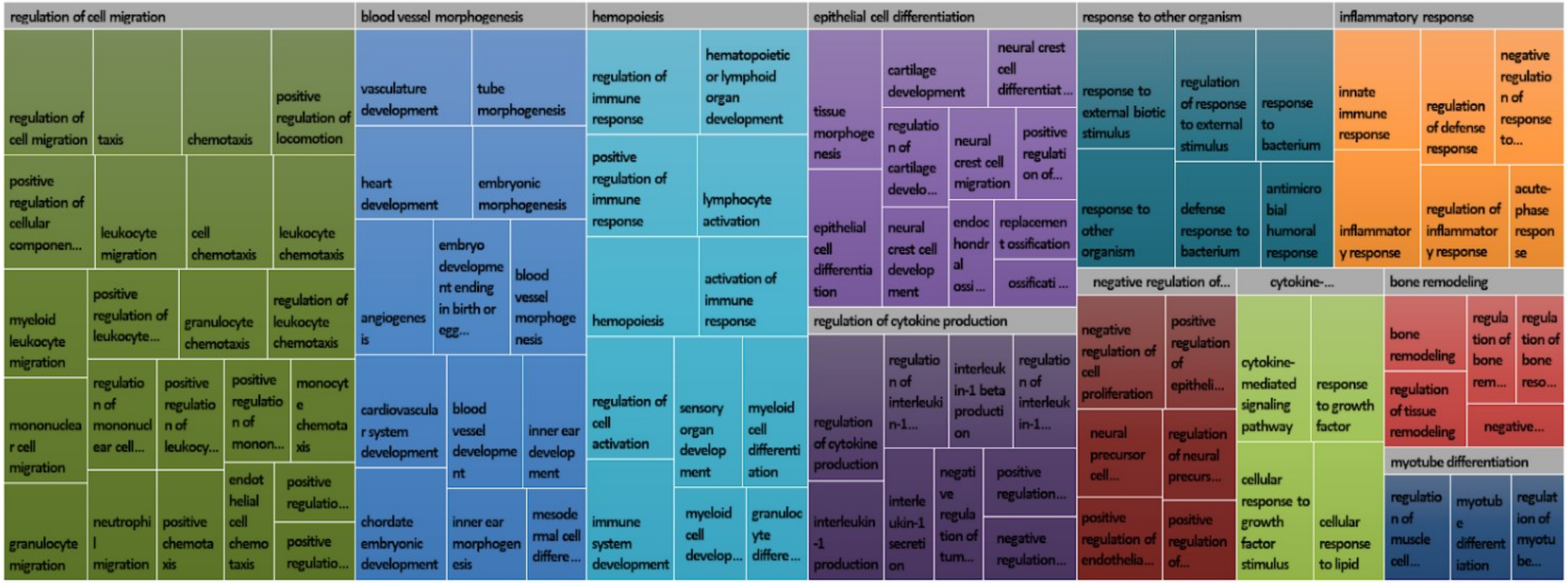

Supplement: Supplementary file 1 [file genes-11-01106-s001.zip › FigureS7.pdf]
