# Supplementary material for: Unveiling Sex-Based Differences in the Effects of Alcohol Abuse: A Comprehensive Functional Meta-Analysis of Transcriptomic Studies
Source: Genes (Basel). 2020 Sep 21;11(9):1106. doi: 10.3390/genes11091106 (PMC7564639; doi:10.3390/genes11091106)
Supplement: Supplementary file 1 [file genes-11-01106-s001.zip › TableS1.pdf]

Table S1. Software and versions used in this work.

Software / R package	Version
R	3.5.1
affy	1.62.0
annotate	1.62.0
AnnotationDbi	1.44.0
Biobase	2.42.0
biomaRt	2.38.0
dplyr	0.7.8
edgeR	3.24.3
GEOQuery	2.50.5
ggdendro	0.1-20
ggplot2	3.1.0
ggpubr	0.2
GO.db	3.8.2
hgu133plus2.db	3.2.3
hugene10sttranscriptcluster.db	8.7.0
illuminaHumanv4.db	1.26.0
KEGG.db	3.2.3
limma	3.38.3
mdgsa	1.14.0
metafor	2.1-0
metap	1.1
methods	3.5.3
org.Hs.eg.db	3.7.0
pd.hugene.1.9.st.v1	3.14.1
tidyr	0.8.3
UpSetR	1.3.3
utils	3.5.3

Metafun-AUD web tool is freely available in https://bioinfo.cipf.es/metafun-aud
